# Evaluating the efficacy of a web-based self-help intervention with and without chat counseling in reducing the cocaine use of problematic cocaine users: the study protocol of a pragmatic three-arm randomized controlled trial

**DOI:** 10.1186/s12888-015-0518-6

**Published:** 2015-07-10

**Authors:** Michael P Schaub, Larissa J. Maier, Andreas Wenger, Lars Stark, Oliver Berg, Thilo Beck, Boris B. Quednow, Severin Haug

**Affiliations:** Swiss Research Institute for Public Health and Addiction at the University of Zurich, Konradstrasse 32, P. O. Box, 8031, Zurich, Switzerland; Arud, Centres for Addiction Medicine, Konradstrasse 32, 8005 Zurich, Switzerland; Department of Psychiatry, Psychotherapy and Psychosomatics, Clinical and Experimental Pharmacopsychology, Psychiatric Hospital of the University of Zurich, Lenggstrasse 31, 8032 Zürich, Switzerland

**Keywords:** Cocaine, Internet, Chat, Web-based, Self-help, Cognitive behavioral therapy, Motivational interviewing, Social cognition

## Abstract

**Background:**

Web-based self-help interventions that aim to reduce problematic substance use are able to reach “hidden” consumer groups in the general population who often fear stigmatization and thus avoid institutional addiction treatment. In Western European countries, including Switzerland, cocaine is the most widely used psychoactive substance after alcohol, tobacco, and cannabis. Although approximately one in six users develop serious problems of dependency, only a minority seeks help from psychiatrists or in outpatient counseling centers or psychiatric hospitals. Offering web-based therapy treatment may potentially reach users who hesitate to approach institutional treatment services and help them reduce their cocaine use before they get into more serious trouble.

**Methods/design:**

The study will use a three-arm randomized controlled trial (RCT) design to test the efficacy of a web-based self-help intervention with or without guided chat counseling compared with that of a waiting list control condition in reducing or stopping cocaine use. The primary outcome measure will be the weekly quantity of cocaine used. Secondary outcome measures will include the number of cocaine use days in the past 30 days, the severity of cocaine dependence, the use of alcohol, tobacco, and/or other illicit drugs, changes in mental health symptoms, and treatment retention. The self-help intervention will consist of eight modules that are designed to reduce cocaine use and depression symptoms. These modules are based on the principles of Motivational Enhancement Therapy and Cognitive Behavioral Therapy, such as Behavioral Self-Management. The three individual chat therapy sessions will be based on the same therapy approaches and will be tailored to participants’ self-help data and aim to assist the reinstatement of social rewards and the improvement of social support and relationships.

**Discussion:**

This study will be the first RCT to test the effectiveness of a web-based self-help intervention in combination with or without chat counseling in reducing cocaine use. The expected findings will contribute substantial knowledge that may help design effective guided and unguided web-based treatment for cocaine users. Moreover, the study will elucidate to what extent a therapeutic alliance with cocaine users can be established in a guided Internet-delivered setting. Additionally, the present study will investigate changes in social support with specific guided therapy interventions that aim to ameliorate social support and social perceptions and compare these changes with those in an unguided self-help intervention

**Trial Registration:**

Current Controlled Trials ISRCTN12205466. Registered 24 February 2015.

## Background

Although the prevalence rates of problematic cocaine use and cocaine dependence are unclear, the lifetime prevalence of cocaine use has almost doubled in the Swiss general population (aged 15–24 and 25–34 years) over the past ten years [1], which is in line with findings in many other countries in Southern and Western Europe [2]. However, the prevalence of cocaine use might be underestimated in general population surveys [3, 4]. Since 2005, resident institutions and outpatient units for the treatment of substance use disorders have reported that cocaine is more or less equally common as opiates in terms of the main problem substance upon entry into treatment [5].

It is assumed that quasi-controlled, occasional use of cocaine is much more prevalent than addicted use and that only a few users are currently in treatment [6]. Nevertheless, some cocaine users switch from controlled consumption to more problematic consumption—in the sense of use that is harmful to oneself or others—and to cocaine dependence [7]. Previous research has estimated that about one in six users who have used cocaine at least once will develop cocaine dependence [8]. For problematic users and those with dependence symptoms, appropriate interventions follow the Concurrent Cover principle (i.e., minimally invasive, low-cost interventions that enhance therapeutic intensity according to need).

Outpatient treatment that is based on *Cognitive Behavioral Therapy* (CBT) has been successful in treating cocaine dependence, while intense research on psychopharmacological approaches over the past three decades has not identified any compelling evidence of effective medicinal treatments [9]. Nevertheless, Swiss narcotics law allows the off-label use of prescription drugs, e.g., methylphenidate or modafinil, to treat the symptoms of cocaine dependence. However, health insurance coverage for such treatments is not guaranteed [9]. Further treatment approaches, such as *Contingency Management* or the *Community Reinforcement Approach*, which reward cocaine abstinence with money or use positive reinforcement, have primarily been reported in the United States and seem hardly applicable to European countries due to health care policy or health insurance-related differences [9, 10].

Web-based interventions that aim to reduce problematic cocaine use might fill an important gap by providing support for problematic cocaine users or those with the initial dependence symptoms who have not responded well to the present institutional treatment offerings. The Internet is a useful tool to reach hidden populations, such as illicit drug users [11]. Furthermore, web-based interventions are easy to access and show a remarkably positive cost-benefit relationship [12], which is an important advantage for Switzerland and other industrialized countries with increasing health care costs.

However, web-based interventions have primarily focused on reducing alcohol and cannabis use [13–17]. Web-based interventions to reduce problematic stimulant drug use are sparse and report mixed results [18, 19]. The first meta-analysis on the effectiveness of web-based interventions in reducing alcohol use yielded encouraging results [20]. The most promising approaches for web-based interventions that aim to reduce substance use are based on CBT, including *Behavioral Self-Management* (BSM), relapse prevention [21, 22] and *Motivational Enhancement Therapy* (MET) [19]. Web-based self-help interventions with guided individual CBT chat counseling were found to reduce alcohol use and alcohol use disorder symptoms in problematic alcohol users [23]. Similar positive outcomes have been shown in guided individual CBT for (subclinical) depression and anxiety disorders [24, 25]. Therapeutic alliance has been shown to reach high levels in the guided web-based individual cognitive-behavioral treatment of depression, generalized anxiety and social anxiety disorders, and post-traumatic stress disorder, with mixed evidence of its impact on the respective main outcomes [26–28]. The therapeutic alliance in guided real-time web-based CBT has not been investigated in the frame of preclinical or clinical substance use disorders, which could be of importance in the light of recent findings on social cognitive deficits in cocaine users. Problematic cocaine users might possess rather universal social-cognitive deficits and decreased social networks, depending on the frequency of use, compared with stimulant-naïve controls [29, 30]. It has been argued that basic social interaction deficits in cocaine users may arise from altered social reward processing [31].

In a previous study, we demonstrated the feasibility and potential effectiveness of the initial web-based self-help intervention, Snow Control (without chat), which was designed to reduce problematic cocaine use [18, 32]. Overall, treatment retention was very low. However, participants in the self-help intervention showed increased treatment retention compared with the psycho-educative control group. The factors that contributed to subject treatment retention included the low severity of cocaine dependence, age, and depression symptoms. The average number of cocaine use days per week did not change substantially, whereas the weekly quantity of cocaine use was reduced equally in both groups. Many participants set very moderate consumption goals and quit the program after achieving their rather low goals in 1–2 weeks [18]. Therefore, a thorough revision of this preliminary self-help intervention was developed; this revision considers the need for more structured goal setting, more personalized advice, and sustained motivation and accounts for potential depression symptoms with CBT for depression therapy [33] and social problem solving [34]. In addition, this revised version offers guided anonymous chat counseling to foster socially rewarding and non-cocaine-related contacts and relationships.

The present study aims to investigate and compare the effectiveness of the revised web-based self-help intervention, Snow Control 2.0, with tailored chat counseling that is based on CBT, MET, BSM, and social problem solving in reducing problematic cocaine use. More specifically, a three-arm randomized controlled trial (RCT) will be conducted to test the effects of a self-help intervention with and without chat counseling compared with those of a waiting list control group.

### Study interventions

**Fig. 1:**
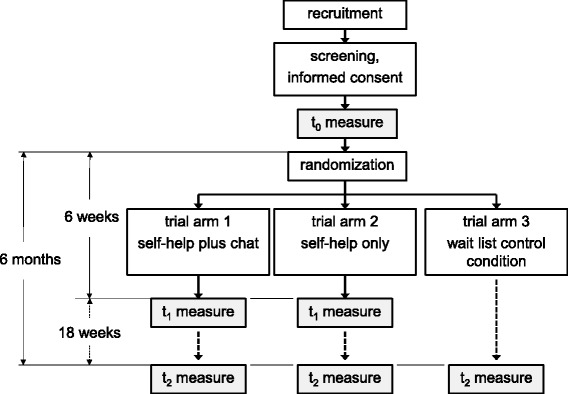
Trial flowchart: an overview of the participant flow for this three-arm randomized control trial

The first-arm intervention consists of three individual chat-counseling sessions that are based on MET and CBT and the web-based self-help intervention from study arm 2. These chat sessions are tailored to the data that are derived from participants’ self-help interventions. Chat counseling will explore the currently available social support for cocaine users, help improve their valuable relationships, and respond to their individual requests. The web-based self-help intervention from study arm 2 is based on classical CBT for the treatment of cocaine dependence [1], BSM [21], MET [35], CBT for depression [33], and social problem solving [34]. Study arm 3 consists of a waiting list control condition. Figure 1 presents a detailed overview of the aforementioned study arms.

#### Self-help intervention modules

The web-based self-help intervention (study arms one and two) is organized in two parts. Part 1 can be accessed after successful registration and completion of the baseline assessment. Part 1 has to be worked through systematically. Part 2 is presented in a clearly arranged menu (see Fig. 1), and its modules are freely accessible as soon as part 1 is completed. Nevertheless, we recommend working through the modules in the order presented below, as long as no specific module is indicated (e.g., strong craving). The participants are allowed to repeat the modules whenever needed.

- Part 1: Introduction
  - o Registration process
  - o Personal companion (an introduction of 6 companion profiles and the participant’s personal selection of his or her companion)
  - o Examination of the pros and cons of changing cocaine consumption patterns to address motivation
  - o Goal setting and introduction to e-mail reminders
  - o Introduction to the cocaine consumption diary and its fully automated progress charts and statistics
  - o Introduction to the “My Snow Control” folder: individuals can review their acquired module documents (e.g., the list of the top five strategies for managing cocaine cravings)
  - o Introduction to the emergency button for immediate responses to frequently asked questions and access to emergency contacts
- Part 2: Modules
  - o Module 1: Strategies for goal achievement
  - o Module 2: Identifying your risk situations
  - o Module 3: Caring about your needs: reduction of stress and depression symptoms and sleep hygiene
  - o Module 4: Managing cocaine cravings
  - o Module 5: Managing relapses
  - o Module 6: Six-step program to tackle your problems
  - o Module 7: Say “no” to bolster refusal skills
  - o Module 8: Preserving achievements

**Fig. 2:**
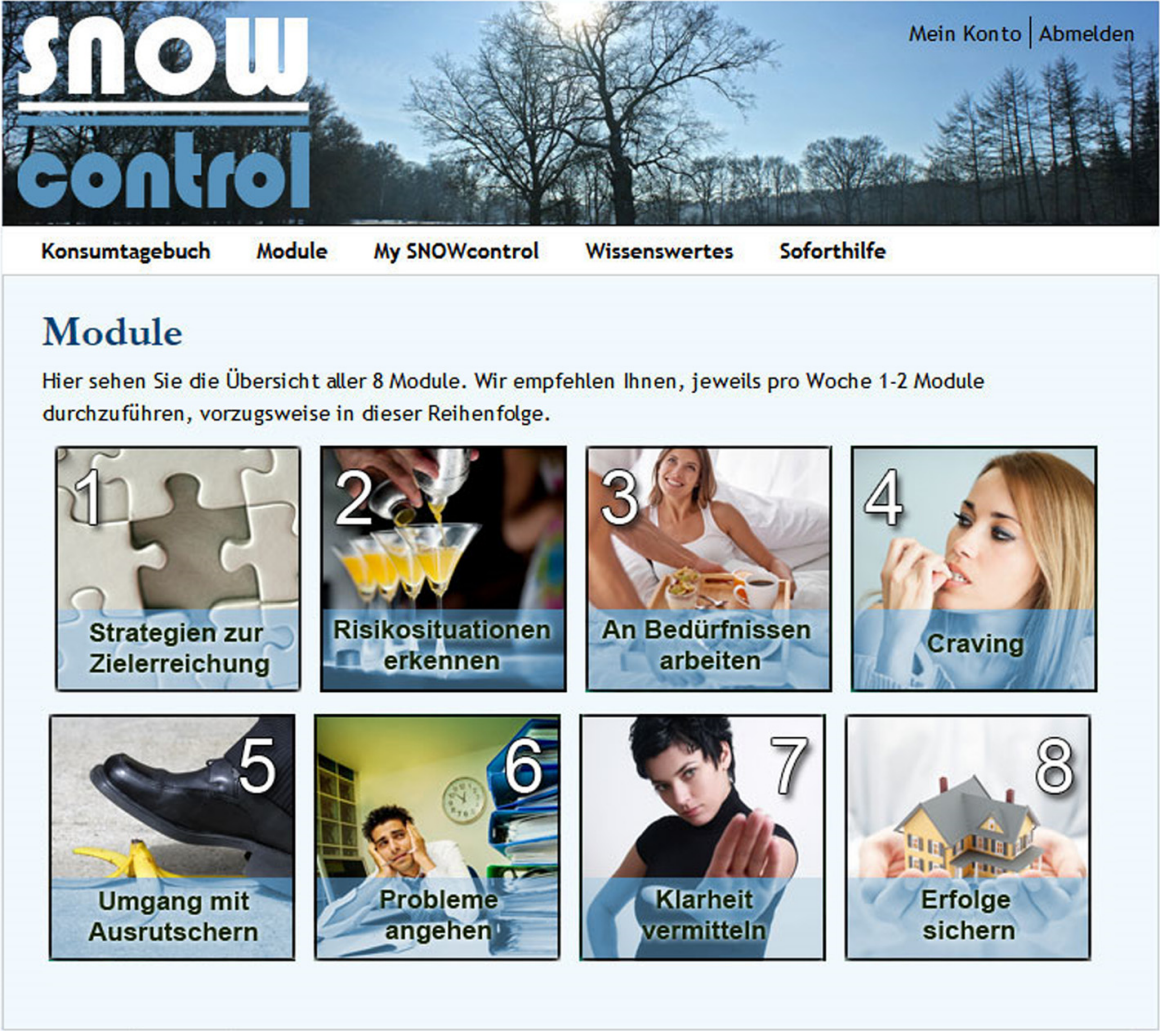
Trial flowchart: an overview of the participant flow for this three-arm randomized control trial

Fig. 2 Main menu of the self-help study arm including the chat-window that is visible in the self-help plus plus chat study arm only. Furthermore, a glossary that explains the terms, definitions, and concepts that are used in the intervention will be provided in an appendix that can be accessed after registration. This glossary will contain information about the history of cocaine use, the short-, medium- and long-term effects of cocaine use, the physical risks of cocaine use (e.g., addiction, cardio-vascular diseases), and co-occurring mental health problems (e.g., depression, psychosis). Moreover, frequently asked questions and their corresponding answers will be presented.

#### Personal companion

At the beginning of the self-help intervention, six companions will be briefly introduced. Study participants choose a companion with whom they can best identify themselves. The characters vary with regard to gender, age, sexual orientation, family status, and professional circumstances to provide maximum identification. In each module, the personal companion may provide specific advice or examples of useful intervention strategies. Furthermore, the participants may switch between different personal companions to gather additional advice and examples.

#### Goal setting and motivation-enhancing e-mail feedback

Additional tailored motivational advice will be developed and implemented to prevent participants in study arms one and two from setting their consumption goals too low and thus quitting the intervention within the first couple of weeks, as frequently occurred in the preliminary Snow Control study [18], or from setting their goals too high and risking early relapse. Participants will be encouraged to set their overall six-week goal in the beginning and to envision reducing their weekly cocaine use by at least 40 % over the course of the six-week intervention. In a second step, tailored weekly instructions will be provided, which encourage a successive weekly reduction of 20–30 % in the first three weeks. If the planned reduction goal is not met, motivational enhancement will be set to a more realistic goal (e.g., 15–20 %). The participants who seek cocaine abstinence will be encouraged to make similar step-by-step reductions until full abstinence has been attained. To avoid severe withdrawal symptoms and potential health risks, abrupt cocaine abstinence will not be recommended, as is often recommended when attempting to quit, e.g., smoking or chewing tobacco [36]. Participants who achieve their weekly aims will receive positive feedback and will be encouraged to continue in a similar vein to achieve or even exceed their overall goals. Once participants have achieved their overall goals, they will be encouraged to maintain their reduced cocaine use or abstinence or to further reduce their use successively until week 6. Moreover, weekly motivation-enhancing e-mail reminders will be sent; these e-mails will contain feedback that is tailored to the previous weeks of the intervention and the participant’s module progress.

#### Extensions in the chat condition

The three additional chat counseling sessions (only in study arm 1) will support behavioral changes through MET. Participants in the chat condition can discuss the modules of the web-based self-help intervention and review the development of their consumption diary; they are also motivated to foster and review socially rewarding contacts. Social reward sensitivity is assumed to be limited in the self-help only study arm 2, which does not include sensitizing individual counseling.

The chat sessions will be structured as follows:

- Chat session 1: Starting point and objective agreement
  - o Personalized feedback according to the baseline assessment and potential cocaine-related physical and mental health risks
  - o Review of successful strategies for change that are related to topics other than cocaine
  - o Imagining daily routines, structuring the day and managing boring situations after cocaine cessation/reduction with new rewards; exploring the aim to live with cocaine abstinence or with low cocaine use and the dissonances with its current use; and mobilizing social support and the option of inviting a relevant third party to join chat session 2
  - o Review of and agreement on the overall cocaine use objective
  - o Review strategies to deal with cocaine cravings and perspectives for chat session 2
- Chat session 2: Exchange of experiences, social support, and relationships
  - o Exchange of experiences regarding the agreed-upon objective since the beginning of the intervention
  - o Review of the patterns that relate to the consumption diary goals and achievements
  - o Review and consolidation of web-based self-help modules
  - o Review of social rewards prior to cocaine use
  - o Experience and improvement of existing social support and relationships
  - o Revision of intervention objectives and perspectives for chat session 3
- Chat session 3: Review and consolidation
  - o Exchange of experiences regarding the agreed-upon objective since the beginning of the intervention
  - o Review of the patterns that relate to the consumption diary goals and achievements
  - o Consolidation of web-based self-help modules and identification of the best five strategies for long-term success
  - o Experience of intention to change and improvement of social support and relationships
  - o Definition of long-term objectives and consolidation of strategies for long-term success

Health care professionals will be assigned as online counselors, who will contact registered users during their 6-week programs to arrange three chat counseling sessions. To conveniently manage their interactions with clients, counselors will have access to a specific user management area to add arranged chat dates, define current statuses, and add personal comments about their clients. With this tool, counselors can follow their clients’ progress in reducing their cocaine use through clearly arranged charts, monitor their clients’ module progress, and look up previous chat histories. Specific lists will help counselors track their clients (e.g., a list with “all users”, “my clients”, or “my upcoming chat sessions”). All written dialogues during chat counseling sessions will be recorded in a database for further analysis.

#### Waiting list control condition

Participants who are randomized to the waiting list will have the opportunity to participate in the web-based self-help intervention 6 months after registration. Follow-up measures for these participants will be assessed online or through telephone interviews for those participants who cannot be reached online at the 6-month follow-up.

#### Technical specifications

Snow Control 2.0 is a website that is based on Drupal 7, a content management system with a responsive design for computer screens, tablets and smartphones.

Any Internet user can register an online account via www.snowcontrol.ch by providing a unique username and e-mail address. Registered users will be asked to take care to not disclose any personal information about their real identities. For this purpose, they will be advised to register an anonymous e-mail account with a third-party e-mail provider. To prevent the creation of accounts with invalid e-mail addresses, the registration process will not be complete until a verification e-mail link has been clicked and a personal password been set.

## Methods/design

### Aims of the trial

In a three-arm RCT, the current study aims to compare the effectiveness of three interventions—guided chat-counseling and web-based self-help (arm 1), web-based self-help alone (arm 2) and a waiting list control condition (arm 3)—in reducing cocaine use in problematic cocaine users. This study will be conducted following the CONSORT-EHEALTH (Consolidated Standards of Reporting Trials of Electronic and Mobile HEalth Applications and onLine TeleHealth) who are an extension of the CONSORT statement [37, 38]. The primary outcome measure is the weekly quantity of cocaine used at the 6-month follow-up. The secondary outcome measures include the number of cocaine use days over the past 30 days, the severity of cocaine dependence, the use of other psychoactive substances, mental health symptoms, social support, intervention satisfaction, and treatment retention. Furthermore, client satisfaction will be measured in study arms 1 and 2. Moreover, the working alliance between the online counselor and the participants in the chat conditions will be explored. Finally, the predictive validity of participant characteristics for treatment retention and primary and secondary outcomes will be examined.

### Study population

The study population will be recruited through the revised Snow Control 2.0 website, several websites from local outpatient treatment centers, and websites with nightlife prevention offers. In addition, advertisements will be placed in Internet-forums and (commuter) newspapers. Moreover, we plan to send mailings, including flyers, to local addiction counseling centers, general practitioners, psychotherapists and psychologists.

### Hypotheses

We hypothesize that web-based self-help interventions, which involve interactions with real counselors, will be more effective in reducing cocaine use among problematic cocaine users compared with self-help interventions only and the waiting list condition. We will test the following detailed study hypotheses with respect to the main outcome, i.e., a reduction in the weekly quantity of cocaine used between the baseline assessment and the 6-month follow-up:

1. *In reducing problematic cocaine use, tailored chat counseling in combination with the web-based self-help intervention (study arm 1) is more effective than the waiting list control condition (study arm 3).*
2. *In reducing problematic cocaine use, the web-based self-help intervention (study arm 2) is more effective than the waiting list control condition (study arm 3).*
3. *In reducing problematic cocaine use, chat counseling in combination with the web-based self-help intervention (study arm 1) is more effective than the web-based self-help intervention alone (study arm 2).*

Analogous hypotheses apply to the secondary outcomes, including the severity of cocaine dependence, alcohol and other drug use, and mental health symptoms, of study arms 1 and 2 and the waiting list control group. Moreover, we have the following specific expectations:

1. *Participants in study arm 1 will demonstrate increased social support at the 6-month follow-up compared with those in study arms 2 and 3.*
2. *After 6 weeks of intervention, participants in study arm 1 will be more satisfied overall with the received intervention than those in study arm 2.*
3. *Participants in study arm 1 will show higher intervention retention rates than those in study arm 2.*
4. *Participants in study arm 1 will show greater self-help intervention adherence than those in study arm 2.*
5. *Increased working alliance ratings will predict better outcomes for participants in study arm 1.*

### Outcome measures

The primary outcome measure is the weekly quantity of cocaine used, a very promising measure for this type of intervention in problematic cocaine users [18]. Moreover, the following secondary outcome measures will be assessed: (1) The number of cocaine use days over the past 30 days will be calculated using the timeline follow-back assessment [39]. (2) The Severity of Dependence Scale (SDS) is a 5-item questionnaire that will provide a score that indicates the severity of cocaine dependence [40]. (3) The “Fragebogen Substanzanamnese” (FDA), or Substance History Questionnaire, will ascertain the lifetime and past month’s prevalence of use and patterns of use, according to ICD-10. This measure was derived from the European Addiction Severity Index (EuropeASI) [41]. (4) The short version of the Mental Health Inventory (MHI-5) [42], which is a validated and user-friendly self-assessment questionnaire, will assess recent mental distress and self-reported diagnoses of depression. (5) The Social Support Questionnaire (SSQ-6) is a 6-item outcome measure that uses a six-point Likert scale, ranging from 1 (very dissatisfied) to 6 (very satisfied), to assess the number of persons that users can count on for help, support, consolation and other social support and how satisfied they are with that support system [43]. (6) The Working Alliance Inventory (WAI-SR) is a 12-item self-report questionnaire that consists of three subscales that are designed to assess three primary components of the working alliance [44]: (i) how closely the client and therapist agree on and are mutually engaged in the goals of treatment, (ii) how closely the client and therapist agree on how to reach the treatment goals, and (iii) the degree of mutual trust, acceptance, and confidence between the client and therapist. The composite score is used as a global measurement of working alliance. (7) Client satisfaction with the intervention will be investigated using the Client Satisfaction Questionnaire (CSQ-4), a brief user-friendly instrument with good psychometric properties that has been tested in numerous studies with diverse client samples [45]. In study arms 1 and 2, retention will be assessed by the last entry in the consumption diary. Finally, in study arms 1 and 2, participation in the self-help intervention will be measured through diary entries, the number of completed modules, and the number of logins in the login history between the baseline assessment and the 6-month follow-up. Table 1 depicts an overview of the involved outcome measures.

**Table 1:** Overview of measurements and instruments

Assessments/instruments	Baseline	6 weeks	6-month follow-up
Socio-demographics	x		
SDS	x	x	x
Quantity of cocaine use^1)^	x	x	x
Days of cocaine use over past 30 days	x	x	x
FDA	x	x	x
MHI-5	x		x
SSQ-6	x	x	x
WAI-SR^2)^		x	
CSQ-4^3)^		x	

^1)^ The weekly quantity of cocaine use will be derived from the consumption diary and measured every week between the baseline assessment and week 6

^2)^ This instrument will only be applied to participants in study arm 1 (the self-help intervention with chat counseling)

^3)^ This instrument will only be applied to participants in study arms 1 and 2 (self-help intervention with chat counseling vs. self-help intervention alone)

### Estimation of the expected effect sizes and sample size

Based on the results of our previous study [18], we expect small to medium effect sizes of at least 0.30 (Cohen’s d) between t0 and t2 when we compare the amount of cocaine used in participants in study arm 2 (web-based self-help intervention without chat counseling) with those in study arm 3 (the waiting list control). Assuming that study arm 1 (web-based self-help intervention with chat counseling) will result in a larger Cohen’s d than study arms 2 and 3, we rely on the aforementioned comparison (arm 2 vs. arm 3) for sample size estimation. A sample size of *n* = 144 in each study group would reveal 80 % power (F-test, df = 2, α = 5 %) to detect this difference based on calculations performed with G-Power software. Thus, we aim to recruit a total of 432 study participants.

### Consent procedure

The Snow Control 2.0 webpage will explain the study rationale to the participants. The participants will then be informed of the following: (1) study inclusion and exclusion criteria (see Table **2**); (2) the three different arms and their 1:3 chance of being assigned to one of the arms; (3) the potential risks of participation; (4) safety arrangements during and after the study phase; (5) the inability of Snow Control (with or without chat counseling) to replace face-to-face therapy for problematic cocaine use; and (6) the circumstances that will require them to contact their general practitioner or another medical professional. Moreover, the emergency list will be introduced and will be accessible throughout the intervention via an emergency button. The participants will also be informed that the ethics committee of the Canton of Zurich has approved the study. Furthermore, participants will be informed about their right to withdraw from the study at any time without any negative consequences, except for the loss of study compensation. Informed consent is provided when participants have read the informed consent page and submitted their consent by clicking a submission button.

**Table 2:** Inclusion/exclusion criteria and reasoning

Inclusion criteria	Reasoning
Minimum age of 18 years	To ensure a minimum age for participation
Cocaine use > 3 occasions in the past 30 days	To also include occasional users to provide extended study validity
Exclusion Criteria	Reasoning
Participation in other psycho-social or pharmacological treatments for the reduction/cessation of cocaine use	To avoid confounding treatment effects
Opioid use during the past 30 days (exception: substitution maintenance treatment for opioid dependence without heroin use in the past 30 days)	To avoid confounding drug effects
Previous treatment for cardiovascular problems or apoplexy	To avoid that subjects who have these health problems

### Baseline assessment

Participants who do not meet the requirements (see Table 2) will not be permitted to start with the baseline assessment or participate in the program. Those participants who meet the requirements will be forwarded to the baseline assessment (see Table 1). The completion of the baseline assessment will allow participants to begin their study arm according to an automated online allocation procedure.

### Randomization and allocation

After they have completed their baseline assessment, participants will be randomly assigned in a 1:1:1 ratio to 1 of the 3 study arms. As we offer full transparency on the three study arms, we expect a certain risk that some participants might register another account in an effort to change their assignment and access a different study arm. In that case, the participant will remain in the initially assigned study arm for the rest of the day, based on his or her IP address.

### Safety

During the 6-week intervention, participants are provided with an emergency button for immediate responses to frequently asked questions, access to emergency contacts, and the telephone number of a collaborating outpatient clinic in a nearby city. Participants will be informed about how to use these contacts. Access to emergency information will be granted to participants before, during, and after the study until the 6-month follow-up, even if they withdraw from the study.

### Experience, training and supervision of chat counselors

To ensure that chat counseling sessions in study arm 1 are conducted with the same MET style and cover all relevant points, the chat counselors will be trained MET counselors with at least one year of experience in treating patients with cocaine use-related problems, and each counseling session will follow a checklist. In a first step, two senior psychotherapists from the “Arud” centers for addiction medicine, who have extended face-to-face and web-based addiction counseling experience, will be trained. Furthermore, they will supervise junior therapists after a thorough self-experience phase with extended supervision. Specific addiction chat counseling quality standards will be developed and implemented for this study based on the EQUS treatment quality standards [46] and the Swiss national addiction counseling portal quality standards [47].

### Trial flow

Figure 1 provides an overview of the trial flow. If participants successfully complete the baseline assessment (t0), they will be introduced step-by-step to the corresponding study arm. Participants will be invited to participate in part 1 (study arms 1 and 2) or informed that they will be provided with access to the web-based self-help intervention in 6 months (waiting list condition). Participants in intervention arms 1 and 2 will receive automated e-mail notifications to login and report the amount of cocaine they use and their frequency of use in their consumption diaries every week. Access to all modules in part 2 will be unrestricted from day one. However, the participants will be encouraged to complete all modules in the designated order unless there are urgent individual reasons to skip to a specific module. They are encouraged to repeat any modules if they feel the need.

The follow-up assessment will be performed in three steps. First, participants will be invited via e-mail to participate in the assessment. Up to three reminders will be sent, and participants will be informed that completion of the entire 6-month follow-up assessment will automatically register participants in a lottery for a tablet computer with the option of donating the corresponding value to a charitable organization. Should participants fail to complete the 6-month follow-up despite these reminders, they will be contacted via telephone and offered an interview by study collaborators. If participants refuse a telephone interview, they will be offered an interview on the primary outcome only. If participants still refuse their follow-up participation, their reason for denial will be documented.

### Management of intervention reminders

Each week, participants in intervention arms 1 and 2 will receive a motivation-enhancing e-mail that reminds them to login to their consumption diaries. If participants do not fill out their consumption diaries, they will receive 6 additional reminder e-mails every 36 h. If participation is not continued after these reminders, they will no longer receive reminder e-mails. Participants in study arm 1 (chat counseling and self-help intervention) will also be invited to the first chat session at least three times over a two-week period if they do not respond to the first invitation. Each invitation will contain at least three dates for chat sessions, as suggested by their counselor. If they are unable to attend the chat session on any of these dates, participants can suggest dates to their counselor. This procedure will be repeated until a first chat date has been set. If participants do not show up on the agreed chat date, they will be sent a new chat date until the first chat appointment is realized. If participants do not reply after three invitations to schedule chat appointment, they will no longer receive chat requests from their counselor, unless they actively make an effort to contact their chat counselor within 6 weeks of the intervention. The second and third chat appointments will be scheduled at the end of the preceding chat session. If participants do not show up for the subsequent sessions, they will be sent new suggestions for chat dates (twice for each session). Finally, yet importantly, the participant and counselor may terminate the counseling if they agree that it is no longer needed at the end of each chat session.

### Data analysis

Data will be analyzed according to the intention-to-treat principle (ITT). For the ITT analyses, we will apply the multiple imputations procedure (MICE) of STATA, which imputes missing data using all available data on a person. Baseline measurements will be compared using t-tests and chi-square tests. Differences between primary and secondary outcome variables between the baseline and follow-up assessments will be tested using the generalized estimating equation (GEE) models. The GEE model is a repeated-measures regression model that considers the correlations between the repeated measures from each person [48]. We will perform logistic GEE analyses for the binary outcome variables and linear GEE analysis for the continuous outcome variables. The results from the imputed data set will be crosschecked with the non-imputed data set.

We will conduct additional exploratory regression analyses to determine whether baseline variables predict the frequency and quantity of cocaine use, the severity of cocaine dependence (SDS), reduced psychiatric symptoms (MHI-5), social support (SSQ-6) at follow-up and treatment retention. For these analyses, we will use linear, multinomial, or binary regression models, depending on the scale of the outcome measure. Similar regression analyses will be conducted for working alliance (composite and subscale WAI-SR scores) in relation to primary and secondary outcomes for the participants who receive the additional chat counseling in study arm 1 and for client satisfaction (CSQ-4) sum score in study arms 1 and 2. The treatment retention and client satisfaction of study arms 1 and 2 will be compared using chi-square tests at week 6 (intervention end).

### Ethical review

This RCT will be executed in compliance with the Helsinki Declaration and has been approved by the ethics committee of the Canton of Zurich on February 2, 2015 (KEK-ZH-No. 2014–0611).

## Discussion

To the best of our knowledge, this study will be the first RCT to test the effectiveness of a web-based self-help intervention with and without chat counseling in reducing problematic cocaine use. This study will have important implications for the ongoing discussion about whether interactive web-based interventions with personal contacts is more effective in reducing substance use in problematic users than individual self-help interventions. Compared with the previous Snow Control web-based self-help intervention [18, 32], we have added a step-by-step goal setting procedure that is based on MET. Moreover, numerous interactive conditional and personalized contents have been included in the modules and motivation-enhancing e-mail feedbacks that are tailored to the cocaine reduction progress in the consumption diary have been added. In addition, this study will investigate to what extent and for which participants the development of a therapeutic alliance in the applied chat counseling setting (arm 1) is possible. More precisely, this study will explore the impact of the therapeutic alliance on substance use outcomes, as in previous research that has investigated the effects of web-based CBT on depression, generalized anxiety and social anxiety disorders and post-traumatic stress disorder [26–28]. We will also explore potential differences between the three trial conditions in terms of how social support during the intervention affects the reduction of cocaine use.

To confirm the effectiveness of the self-help intervention and chat counseling, these parts will be translated and implemented in Safe Zone, a Swiss addiction portal [47]. Thus, this intervention has a high likelihood of helping many problematic cocaine users, according to the principle of concurrent cover, in a very cost-effective way. Especially well-integrated cocaine users who worry about stigmatization and thus do not go in for addiction counseling or check themselves into treatment centers may profit from this anonymous intervention [32]. Moreover, users who prefer social distance and rely on after-work evening chat sessions might use such flexible web-based interventions instead of outpatient treatment.

The weekly quantity of cocaine used is the main outcome in the present study. This outcome was very promising in the first Snow Control study [18], as consumption diaries were clearly filled out more than the online questionnaires. Moreover, users are used to counting grams of cocaine [18] because they also buy cocaine in round sums. The street price of 1 g of cocaine is approximately one-hundred Swiss francs. However, as this outcome is rather unusual in the evaluation of brief interventions for the reduction of cocaine use, we will also rely on the number of days that cocaine has been used over the past 30 days.

Apart from active withdrawal of informed consent, we do not maintain any specific criteria for dropouts. We learned from the consumption diaries of earlier studies [16, 18] that a substantial number of participants will take breaks for two or more weeks in the recommended 6-week intervention, e.g., for holidays, and then continue several weeks later. Thus, we have decided against limiting intervention access when participants do not fill out their diaries for several weeks; we will only stop e-mail reminders (arms 1 and 2) and counselor contact (arm 1), both of which can be undone if the corresponding participant resumes the intervention or contacts his or her counselor again.

The greatest challenge for the Snow Control 2.0 trial is the expected strong attrition rate during intervention and low numbers for the follow-up assessment. We will address these issues as follows: (1) We will impute missing data. (2) The baseline assessment requires approximately 20 min, which will attract more motivated participants and prevent the participation of less motivated individuals. (3) By allowing the participants to choose their personal companions, the intervention was more tailored to individual participants. The provision of optional contents allows more in-depth information and exploration when required. Moreover, personalized motivation-enhancing e-mail feedback supports individual progress in reducing problematic cocaine use. (4) Participants who complete the 6-month follow-up assessment will be compensated with 40 Euro. Moreover, participants who complete the 6-month follow-up assessment will be entered in a lottery to win 400 Euro (online voucher or online charitable donation).
