# Erratum to: Evaluating the efficacy of a web-based self-help intervention with and without chat counseling in reducing the cocaine use of problematic cocaine users: the study protocol of a pragmatic three-arm randomized controlled trial

**DOI:** 10.1186/s12888-015-0600-0

**Published:** 2015-09-16

**Authors:** Michael P. Schaub, Larissa J. Maier, Andreas Wenger, Lars Stark, Oliver Berg, Thilo Beck, Boris B. Quednow, Severin Haug

**Affiliations:** Swiss Research Institute for Public Health and Addiction at the University of Zurich, Konradstrasse 32, Zurich, Switzerland; Arud, Centres for Addiction Medicine, Konradstrasse 32, Zurich, 8005 Switzerland; Department of Psychiatry, Psychotherapy and Psychosomatics, Clinical and Experimental Pharmacopsychology, Psychiatric Hospital of the University of Zurich, Lenggstrasse 31, Zürich, 8032 Switzerland

Unfortunately, the original version of this article [1] contained an error. The caption on Fig. 2 was incorrect. The corrected caption should read:
